# When can embryos learn? A test of the timing of learning in embryonic amphibians

**DOI:** 10.1002/ece3.2018

**Published:** 2016-03-12

**Authors:** Evie K. Sehr, Lindsay N. Beasley, Kurtis W. Wilson, Brian G. Gall

**Affiliations:** ^1^ Department of BiologyHanover CollegeHanoverIndiana47243

**Keywords:** *Ambystoma maculatum*, development, embryo, learning, spotted salamander

## Abstract

Learning is crucial to the survival of organisms across their life span, including during embryonic development. We set out to determine when learning becomes possible in amphibian development by exposing spotted salamander (*Ambystoma maculatum*) embryos to chemical stimuli from a predator (*Ambystoma opacum*), nonpredator (*Lithobates clamitans*), or control at developmental stages 16–21 or 36–38 (Harrison 1969). Once exposures were completed and embryos hatched, we recorded the number of movements and time spent moving of individuals in both groups and all treatments. There was no significant difference in number of movements or time spent moving among any of the treatments. The groups that were exposed to predator stimuli and a blank control at stages 36–38 were also tested to determine whether there was a difference in refuge preference or difference in survivorship when exposed to a predator (marbled salamander). There was no difference in survival or refuge preference between individuals; however, all individuals preferred vegetated over open areas regardless of treatment type. We discuss hypotheses for the absence of embryonic learning in this species and suggest it may be the result of the intensity of the predator–prey interaction between the predator, large marbled salamander larvae, and the prey, spotted salamander larvae.

## Introduction

Learning is a critical aspect of life history, yet historically, many studies attempting to understand this mechanism have focused on birds and mammals (Thorndike 1898; Pavlov 1927; Skinner 1953; Thorpe 1958; Marler 1970) typically during juvenile or adult life stages, after the brain is fully developed and the functional basis for learning has been established. However, recent studies have begun to demonstrate that experiences during the earliest possible developmental stages, including embryonic development, can lead to durable modifications in behavior (Hepper and Waldman 1992; Sneddon et al. 1998; Wells and Hepper 2006; Mathis et al. 2008; Romagny et al. 2012).

Early developmental stages are typically the most vulnerable stages within an organism's life and are often subject to the highest risk of mortality (Anderson et al. 1971; Orians and Janzen 1974; Stangel 1988). Thus, the earlier an individual can respond to dangerous environmental features, including signals, the greater their probability of survivorship. The acquisition of learned information before birth has a number of implications for the survival and fitness of the organisms in which it is employed. For example, odor learning before birth plays an important role in maternal and kin recognition in a variety of organisms (Hepper 2005). In addition, exposure to food cues in the egg or amniotic sac likely facilitates the learning of preferred food types (Semke et al. 1995; Schaal et al. 2000; Mennella et al. 2001; Wells and Hepper 2006) or may help naïve organisms avoid unpalatable or toxic food (Hudson and Distel 1999).

One particularly strong source of selection on the morphology and behavior of organisms is predation (Lima and Dill 1990). The first study to experimentally test for prenatal learning of predation risk was Mathis et al. (2008). In this study, the authors found that amphibian embryos exposed to chemical stimuli from predators exhibited adaptive modifications in behavior posthatching and thus learned to fear a dangerous predator. In addition, several recent papers have expanded our understanding of this concept and demonstrated that amphibian embryos are capable of relatively sophisticated information gathering and learning in relation to predation risk (Ferrari and Chivers 2009a,b, 2010, 2011; Ferrari et al. 2010a).

Although embryonic learning in amphibians has been tested, the exact timing in which amphibian embryos are capable of this type of learning has not been studied. In mammals, chemosensory learning is theoretically possible when the brain has developed enough to detect a signal in the olfactory receptor, which is then processed by the olfactory bulb (Royet and Plailly 2004). The signal must then be associated with an experience in the amygdala which is then processed by the hippocampus (Royet and Plailly 2004). This occurs in rat fetuses approximately 20 days postfertilization (Stage 35), and these embryos are capable of simple chemosensory learning (Coppola and Millar 1994; see review in Schaal and Orgeur 1992; Smotherman and Robinson 1992). In cuttlefish, chemosensory systems are developed and respond to stimuli 4 weeks prior to hatching, yet rudimentary learning (habituation) is not possible until only 1 week prior to hatching (Romagny et al. 2012). In amphibians, the neural folds fuse around 97 h after fertilization (Harrison 1969). The telencephalon (part of forebrain) consists of two olfactory bulbs and is the receptor for sensory impulses derived from chemical signals received by the olfactory epithelium and vomeronasal organ (Duellman and Trueb 1994). Unfortunately, it is not exactly known when the forebrain and associated structures are sufficiently developed in amphibians to process chemical information and learn. In a study of the brain of *Xenopus*, Eagleson et al. (1995) suggest that the telencephalon increases in size and the olfactory lobe appears after stage 30. Although the first neurites of the olfactory nerve also appear at this stage, the olfactory pit (intermediate between the olfactory placode and olfactory epithelium) does not develop until stage 40 (Klein and Graziadei 1983; Schlosser and Northcutt 2000). This would suggest that chemosensory learning in the egg is not possible until at least this stage and possibly even later.

Previous studies testing for embryonic learning in amphibians have exposed the embryos to cues when the embryos were relatively advanced and close to hatching (Coppola and Millar 1994; Mathis et al. 2008), or, in other cases, the embryos were exposed to stimuli early in development, but were continually exposed to these stimuli until fully developed (Hepper and Waldman 1992; Mathis et al. 2008; Ferrari et al. 2010a; Ferrari and Chivers 2011). In either case, these procedures preclude an analysis of how early this type of learning becomes possible in prenatal amphibians. We commenced an investigation into how early embryonic learning is possible by exposing spotted salamander (*Ambystoma maculatum*) embryos at two different developmental stages to chemical stimuli from a potential predator and observing the posthatching behavior of the larvae.

## Methods

#### Animal collection and maintenance

Spotted salamander (*Ambystoma maculatum*) egg clutches (*n* = 48) were collected from two ponds from a flatwoods near Hanover, IN, between 15 and 17 March 2015. Each clutch had been deposited the previous night, thus minimizing the role of naturally occurring chemical compounds from interfering with the experimental exposure regimen. The clutches were kept in plastic containers (1.23 L) filled with pond water and sealed for transport. Immediately upon arrival at Hanover College, developmental stages were identified using an Olympus SZ61 dissecting microscope at total magnifications ranging from 10× to 20×. A single individual identified developmental stages according to Harrison (1969). Upon arrival, all clutches were found to be between Harrison (1969) stages 2 to 9 (1 to 12 h after deposition). If multiple clutches were transported in a single container, they were separated at the time of identification. Each individual clutch was placed in a plastic container (1.23 L) with approximately 750 mL of deionized water that was conditioned to 14°C (henceforth: conditioned water). The clutches were all placed in an environmental chamber set to 14°C and the positions of the clutches within the chamber were randomized 3 days later. The clutches were monitored daily by observing the developmental stages of 5 haphazardly selected eggs from 5 randomly selected clutches.

Green frog (*Lithobates clamitans*) tadpoles (*n* = 15) and marbled salamander (*Ambystoma opacum*) larvae (*n* = 15) were collected on 16 March 2015 to serve as donors for the nonpredatory and predatory cues, respectively. These were kept at 14°C in individual plastic containers (0.50 L) that were filled with approximately 250 mL conditioned water. The tadpoles were cleaned every other day and fed Chlorella brand micro‐algae mix once every 3 days. Marbled salamander larvae were cleaned biweekly and fed blackworms (*Lumbriculus variegatus*) ad libitum.

#### Treatments

The experimental protocol employed in this study was modified slightly from that by Mathis et al. (2008). Prior to exposing the embryos to chemical stimuli, each clutch was randomly assigned to one of two separate groups to determine the time of exposure, either early (developmental stages 16–21) or late (developmental stages 36–38). In the early developmental stages, the neural folds are elevated (16) and by the end have fused to form the neural tube (21) (see citations above). In the late developmental stages, the embryo is fully formed and blood circulation in the gills occurs (37); the late developmental stages are equivalent to the stages tested by Mathis et al. (2008). In addition, these clutches were then subdivided into three chemical cue treatments: blank control, nonpredator treatment (green frog tadpole), or predator treatment (marbled salamander larvae).

The containers housing the marbled salamanders and green frogs were cleaned 24 h prior to stimulus exposure to ensure adequate accumulation of the appropriate kairomones. The containers were rinsed with conditioned water and 150 mL of conditioned water was then added. Upon each treatment, 50 mL of stimulus water was removed from each container and pooled to reduce variation among individual cue donors.

Fifty milliliters of the respective cue was then slowly administered down the side of the container holding each clutch to minimize disturbance. Two more exposures occurred in 12‐h intervals for a total of three stimulus exposures and 48 h of exposure to chemical stimuli; clutches were not moved during this process. Forty‐eight hours after the initial exposure, the developmental stages of 5 haphazardly selected embryos were recorded from each clutch. The clutches were then gently removed from their containers and rinsed with conditioned water. The containers were additionally rinsed with warm water and conditioned water. Clutches were then housed in approximately 700 mL of fresh conditioned water; this procedure ensured that exposure to the appropriate stimuli was confined to the exposure window. Embryos that fell out of the jelly matrix were preserved in formalin for verification of developmental stages. Latex gloves were changed after cleaning each clutch to minimize the potential of cross‐contamination of chemical cues between clutches.

### Experiment 1 – Larval activity

Experimental chambers were 13‐mL plastic test tubes positioned horizontally with a 5‐mm hole placed approximately 2 cm from the opening of the test tube. Lines were drawn 11 mm apart and a stopper was used to completely close the opening. The experimental chambers were filled with 13 mL of conditioned water. An individual spotted salamander larva, selected haphazardly from a randomly chosen clutch, was inserted into the experimental chamber (*n* ≈ 5 larvae per clutch, *N*_total_ = 156); larvae were tested when the yolk was completely absorbed. The number of lines crossed and the number of movements made by the larva were then recorded for 5 min. At the completion of a trial, the test individual was removed and preserved in formalin and the chamber was rinsed thoroughly with water and the process was repeated. These preserved larvae were later examined with an Olympus SZ61 dissecting microscope, and the tail height and developmental stage were recorded. Modifications in the timing of hatching or the size at hatching have been reported from other amphibians exposed to chemical stimuli from predators (e.g. Sih and Moore 1993; Chivers et al. 2001); however, there was no main effects of age (all *P* > 0.25) or predator exposure (all *P* > 0.6) or interaction (all *P* > 0.08) on either of these variables in this study.

We conducted a two‐way ANOVA for each individual response variable with age of exposure (early or late) and predator treatment (blank, nonpredator, or predator) as the two factors. Assumptions of normality and homoscedasticity were assessed with graphical analysis of the residuals. Both assumptions appeared to be adequately met by these data.

### Experiment 2 – Habitat preference

A second experiment was conducted with recently hatched larvae to determine whether exposure to predator cues during embryological development affected their propensity to seek shelter in aquatic vegetation. Experimental chambers were 2.1‐L containers filled with 2 cm of coarse sand and 0.5 L conditioned water. The chambers were divided into half by a vertical line drawn down the center on one side of the container. One side was randomly selected to contain aquatic plants (*Salvinia* sp.) to provide refuge. The other half of the experimental chamber was left open. A 50‐mL open‐ended plastic tube was placed directly in the center of the chamber. One haphazardly selected spotted salamander larva was inserted into the tube (*n*_total_ = 13, *n*_blank_ = 7, *n*_predator_ = 6) and allowed to acclimate for 10 min. The tube was then slowly removed and the location of the salamander (empty or refuge) was recorded every 30 min for 5 h. The larvae used for this experiment were in the late development exposure group in the blank and predator treatments. None of the larvae had previously been tested in any experiment.

A contingency table and Yate's chi‐squared test, corrected for continuity, were used to determine whether the frequency of observations in the open or in refuge was the same for larvae that were exposed as an embryo to either a control or a predator. We also compared the number of observations in refuge or in the open (for all treatments combined) with a chi‐squared test to determine whether the larvae (irrespective of treatment) spent more time in refuge or in the open.

### Experiment 3 – Larval survival

A final experiment was conducted to determine if spotted salamander larvae that had been exposed as embryos to chemical stimuli from a predator had enhanced survival in actual predation events with this predator. The larvae used for this experiment were in the late development exposure group in the blank and predator treatments. None of the larvae had previously been tested in any experiment. Five larvae from a single clutch (*n*_blank_ = 7, *n*_predator_ = 6) were placed in a 2.1‐L container filled with 1 cm of coarse sand and 1.5 L conditioned water. The larvae were acclimated in the experimental chamber for 24 h. Marbled salamander larvae, which were not fed for a week prior to the experiment, were then individually inserted into each chamber. The number of surviving spotted salamander larvae in each chamber was recorded once every 30 min for 6 h. We used a *t*‐test to compare the total number of surviving larvae in the blank or predator cue treatments at the conclusion of the 6‐h trial.

## Results

### Experiment 1 – Larval activity

We found no significant main effects of either age or predator treatment on the number of lines crossed or the number of movements by recently hatched spotted salamander larvae (Table 1). In addition, there was no significant interaction between these main effects for either response variable (Table 1).

**Table 1:** Mean number (±SE) of movements made by spotted salamanders (*Ambystoma maculatum*) in both the exposure ages, predator treatments, and age × treatment factors; and two‐way ANOVA of the age, predator treatments, and age × treatment factors. There was no significant difference in the number of movements among ages or treatments

Factor	Mean (±SE)	df	*F*	*P*
Exposure age				
Early	1.86 ± 0.23	1	0.575	0.449
Late	1.62 ± 0.22			
Predator treatment				
Blank	1.67 ± 0.30	2	0.077	0.926
Nonpredator	1.82 ± 0.28			
Predator	1.74 ± 0.29			
Age × Treatment				
Early × Blank	1.49 ± 0.36	2	1.030	0.359
Early × Nonpredator	2.00 ± 0.41			
Early × Predator	2.10 ± 0.42			
Late × Blank	1.85 ± 0.37			
Late × Nonpredator	1.63 ± 0.36			
Late × Predator	1.38 ± 0.41			
Residual		211		

### Experiment 2 – Habitat preference

**Figure 1:**
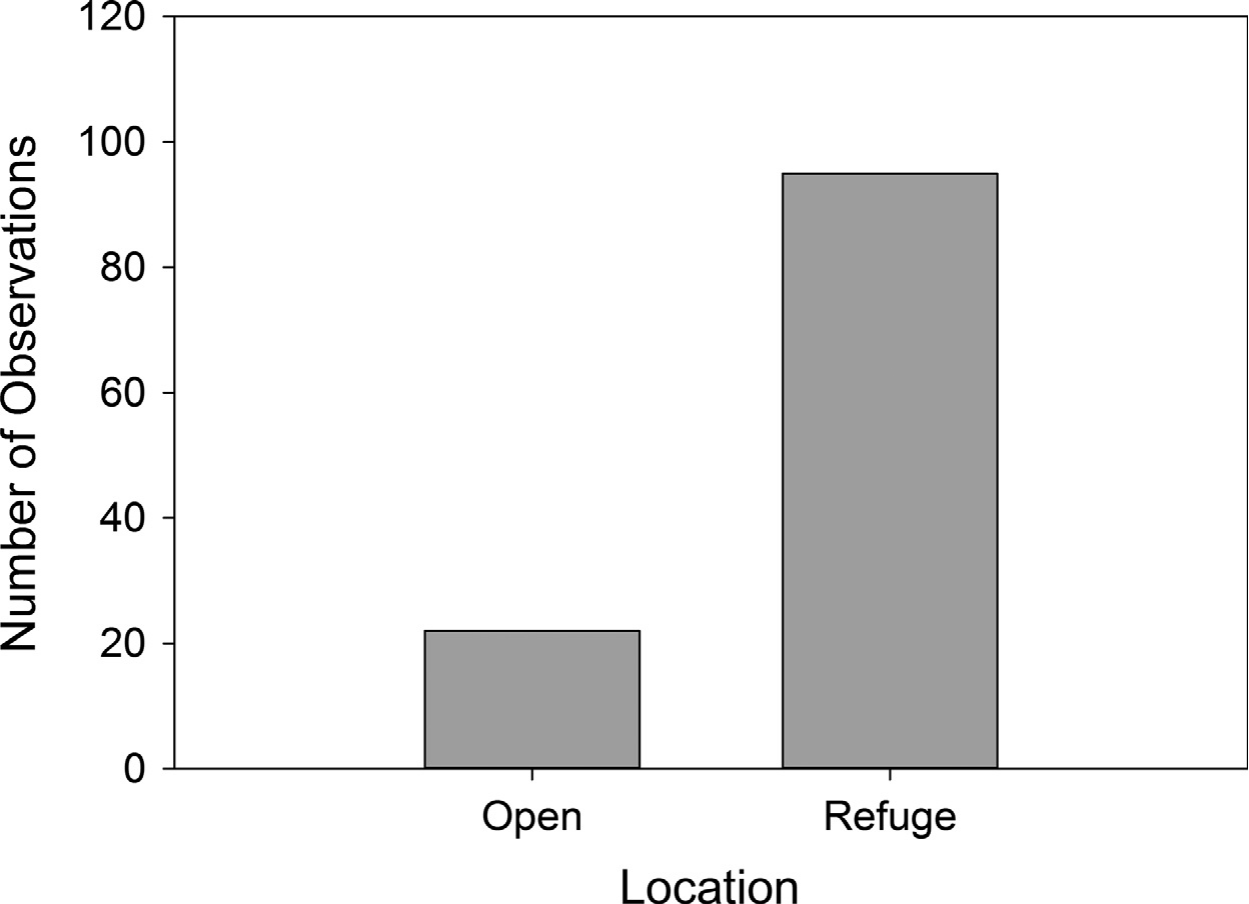
Total observations (*N* = 117) of individuals on each side of the experimental chamber (open or refuge) of both blank and predator treatments. Spotted salamander (*Ambystoma maculatum*) larvae were observed more frequently in refuge (aquatic vegetation) than in open areas of the test arena (df = 1, *χ* ^2^ = 45.5, *P *<* *0.005).

The frequency of observations in the open or in refuge was the same for the two chemical cue treatments (df = 1, *χ* ^2^ = 1.24, *P *=* *0.266, Table 2) demonstrating that individuals exposed as embryos to either predatory chemical stimuli or to a control spent a similar proportion of time in refuge. Additionally, regardless of predator treatment, all larvae were observed more frequently in refuge than in the open (df = 1, *χ* ^2^ = 45.5, *P *<* *0.005, Fig. 1).

**Table 2:** Amount of individuals in each embryo exposure group (blank control and predator cues) tested; number of observations (obs) of individuals on either the open or refuge side of the experimental chamber, as checked every 30 min for 5 h; and *χ* ^2^ between the blank control and the predator cues

Embryo chemical treatment	*N*	# Obs open	# Obs refuge	*χ* ^2^	*P*
Blank control	7	9	54	1.24	0.266
Predator cues	6	13	41		

### Experiment 3 – Larval survival

**Figure 2:**
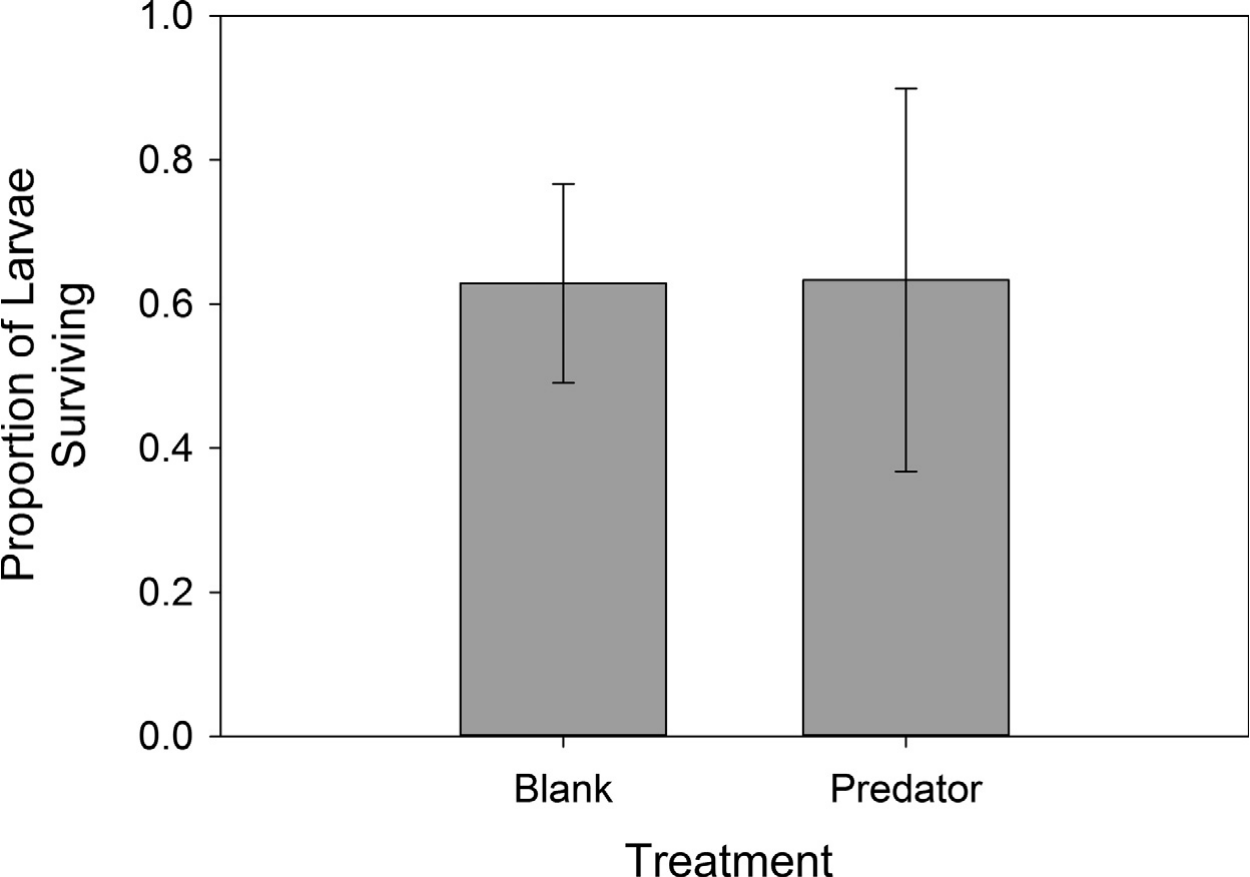
Proportion of spotted salamander (*Ambystoma maculatum*) larvae (in blank and predator treatments) surviving a predatory encounter with a marbled salamander (*Ambystoma opacum*) larvae. There was no significant difference between the two treatments (df = 11, *t *=* *0.042, *P *=* *0.968).

There was no significant difference in the survival of spotted salamander larvae between those exposed as embryos to a blank control and those exposed to cues from a predator during encounters with predatory marbled salamander larvae (df = 11, *t *=* *0.042, *P *=* *0.968, Fig. 2).

## Discussion

In two separate experiments, we failed to find any evidence for learning by spotted salamander larvae that had been exposed to chemical stimuli from predators during two different stages (early or late) of embryonic development. We also failed to identify differential survival of these same larvae in actual encounters with the predator. The negative results with regard to amphibians exposed to predators during the early stages of embryonic development (16–21) are not entirely surprising as the receptors that should be necessary to detect chemical information are insufficiently developed (Harrison 1969; Klein and Graziadei 1983; Eagleson et al. 1995; Schlosser and Northcutt 2000). However, the results with late stage larvae differ from what has been reported in recent studies with other species of amphibians, including another salamander of the genus *Ambystoma*. The first study to test for embryonic learning of predation risk was Mathis et al. (2008). In this study, embryonic wood frogs (*Lithobates sylvatica*) and ringed salamanders (*Ambystoma annulatum*) were exposed to predatory newts and cannibalistic conspecifics, respectively. After hatching, wood frogs showed significant reductions in activity when again exposed to this predatory stimulus. The ringed salamander larvae were also more wary (crossing fewer lines) and spent more time in plants (i.e., refuge) than larvae that had been exposed to control stimuli as embryos. Moreover, several additional studies have documented relatively fine‐scale learning abilities in wood frogs, including the ability to differentiate nonpredators from predators (Ferrari and Chivers 2011) and identify times of day in which a predator is most likely to be foraging (Ferrari et al. 2010a).

Although difficult to explain, there may be several different factors to account for the dramatic differences in results between these studies. The first hypothesis we propose relates to variation in the intensity of risk experienced by our salamander larvae and those tested by Mathis et al. (2008). In this scenario, the level of predation risk the two salamander species are exposed to differs, and this has influenced the evolution of their antipredator behavior. The predation risk allocation hypothesis, first proposed by Lima and Bednekoff (1999), suggests that organisms from a population with a low level of background risk will experience pulses of risk and this variation should lead to these organisms exhibiting strong antipredator defenses. However, if organisms are exposed to high levels of predation risk, exhibiting constant predator avoidance behavior will leave little time for other activities (e.g., foraging), and therefore, these animals should exhibit lower levels of antipredator behavior (Lima and Bednekoff 1999).

The study by Mathis et al. (2008) used cannibalistic conspecifics as a predator in tests of embryonic learning. Cannibalistic morphology in *Ambystoma* is regulated by larval density and food availability (Collins and Cheek 1983; Hoffman and Pfennig 1999; Wildy et al. 2001), both of which can vary dramatically from year to year (e.g., Cecil and Just 1979; Scott 1990; Keith 2009). In ringed salamanders, cannibalistic individuals do not develop different head and teeth morphology as in some other *Ambystoma*, but are simply larger individuals that were deposited as eggs during the early portion of the long breeding season (Nyman et al. 1993). A relatively small proportion of the larvae were large enough to be considered “cannibalistic,” and more importantly, 60% of these large larvae had not consumed conspecifics, but rather ate a variety of alternative prey including cladocerans, copepods, chironomids, beetles, snails, and earthworms (Nyman et al. 1993). The authors suggested that cannibalism may be a highly opportunistic event (Nyman et al. 1993). Several other authors have failed to document cannibalistic feeding in these “large” larvae at all (Trapp 1959; Hutcherson et al. 1989; Kluhsman 1991). These studies suggest that cannibalism may be variable in this species which could lead to more intense antipredator behavior in accordance with the predation risk allocation hypothesis. In contrast, larval spotted salamanders are under intense predation pressure from marbled salamander larvae. This is largely due to the unique life history of this species, whereby females deposit eggs in the fall (~October) which hatch when submerged by increasing water levels in the ephemeral ponds (Petranka 1998). Effectively, marbled salamander larvae have a 2‐ to 4‐month period of growth prior to the influx of resources (i.e., larvae of other species) from spring breeding amphibians, and this species has evolved to take advantage of this resource by becoming a major predator on the eggs and larvae of other amphibians (Petranka 1998). Studies examining the interactions between spotted salamander larvae and predatory marbled larvae have found that spotted larvae are especially vulnerable to predation by this predator compared with other species of *Ambystoma* (Walls 1995). Moreover, field studies have found spotted salamander larvae are exposed to intense predation from these veracious larvae, potentially culminating in the complete elimination from a pond (Stewart 1956; Stenhouse et al. 1983; Stenhouse 1985, 1987). This intense pressure may have led to less overall variation in predation risk, which could lead to lower overall antipredator behavior under the predation risk allocation hypothesis compared to that of ringed salamanders in response to cannibalism.

Another hypothesis for the lack of learned responses in spotted larvae is that the diet of the predator may be important in labeling them as dangerous. It is unlikely the predator in our study had eaten spotted salamander larvae prior to collection because the adults had arrived at the ponds and begun oviposition only a few days prior the collection of eggs. In addition, marbled larvae in our study were fed a benign diet of blackworms for 5 days prior to the collection of their kairomones for the embryonic exposures, thus precluding the presence of dietary cues being present in the kairomones. Predator labeling is common in aquatic environments and occurs in many groups of aquatic invertebrates and vertebrates (Chivers and Mirza 2001; Schoeppner and Relyea 2005; Ferrari et al. 2010b). For example, the marine snail (*Tegula funebralis*) displays predator avoidance behavior in response to kairomones from crabs that have recently fed on conspecifics, but they do not modify their behavior in response to chemical stimuli from crabs that have not eaten or crabs that have been feeding on heterospecifics (Jacobsen and Stabell 2004). In amphibians, red‐legged frogs (*Rana aurora*) and wood frogs (*Lithobates sylvatica*) exhibit similar responses to various invertebrate and vertebrate predators (Wilson and Lefcort 1993; Chivers and Mirza 2001). Unfortunately, additional research is necessary to determine whether predator labeling induces antipredator behavior in spotted salamander larvae.

An alternative to learning to fear threats prior to hatching is to be especially vigilant regardless of prior experience. This innate behavior would be especially important if predation risk is exceedingly high (Bryer et al. 2001), which would result from the ubiquitous nature of the predator and the temporal and spatial stability of the predator–prey interaction. Although we did not find evidence for variation in habitat use by larvae exposed to different predator or nonpredator treatments as embryos, all larvae spent the vast majority of their time in the vegetated side of the test arenas, indicating a high baseline level of predator avoidance behavior. Moreover, previous studies have found that spotted salamander larvae are less vulnerable to predation by marbled larvae when vegetation is available to provide refuge (Brodman and Jaskula 2002). Given that aquatic vegetation is abundant in the ephemeral pools in which spotted salamanders breed, their larvae may not have the capacity to learn to fear these predators due to the ubiquitous nature of the predator and the abundance of refuge.

We failed to find evidence for embryonic learning in spotted salamander larvae in response to potential predators. We also failed to document differential survival between predator‐exposed and control‐exposed larvae during actual predation events. Embryos may lack learned responses due to intense predation risk (predation risk allocation hypothesis), absence of dietary cues in the predators kairomones, or because the prey innately exhibit optimal predator avoidance behavior in the form of hiding in refuge provided by aquatic vegetation.

## Conflict of Interest

None declared.
